# Adipo-Myokines: Two Sides of the Same Coin—Mediators of Inflammation and Mediators of Exercise

**DOI:** 10.1155/2013/320724

**Published:** 2013-06-03

**Authors:** Silja Raschke, Jürgen Eckel

**Affiliations:** German Diabetes Center, Paul-Langerhans-Group of Integrative Physiology, Auf'm Hennekamp 65, 40225 Düsseldorf, Germany

## Abstract

This review summarizes the current literature regarding the most discussed contraction-regulated moykines like IL-6, IL-15, irisin, BDNF, ANGPTL4, FGF21, myonectin and MCP-1. It is suggested that the term myokine is restricted to proteins secreted from skeletal muscle cells, excluding proteins that are secreted by other cell types in skeletal muscle tissue and excluding proteins which are only described on the mRNA level. Interestingly, many of the contraction-regulated myokines described in the literature are additionally known to be secreted by adipocytes. We termed these proteins adipo-myokines. Within this review, we try to elaborate on the question why pro-inflammatory adipokines on the one hand are upregulated in the obese state, and have beneficial effects after exercise on the other hand. Both, adipokines and myokines do have autocrine effects within their corresponding tissues. In addition, they are involved in an endocrine crosstalk with other tissues. Depending on the extent and the kinetics of adipo-myokines in serum, these molecules seem to have a beneficial or an adverse effect on the target tissue.

## 1. Skeletal Muscle and Adipose Tissue as Endocrine Organs

In line with the acceptance of adipose tissue as an endocrine organ [1–3], path-breaking work during the last decade demonstrated that skeletal muscle is an active endocrine organ releasing myokines, which might in part be responsible for the beneficial effect of exercise [4–6]. These myokines are described to communicate with cells in an autocrine/paracrine manner, locally within the muscles, or in an endocrine fashion to distant tissues.

Obesity in a combination with a lack of exercise is a strong risk factor to develop metabolic diseases and type 2 diabetes. Physical inactivity causes the accumulation of visceral fat and the health consequences of both are related to systemic low-grade inflammation [7, 8]. Adipocytes from obese patients are characterized by altered endocrine function, leading to increased secretion of proinflammatory adipokines, such as TNF*α*, chemerin, MCP-1, dipeptidyl peptidase 4 (DPP4), and others [9–14]. Thus, the dysregulation of adipokine secretion is related to metabolic diseases. The activation of inflammatory pathways leads to insulin resistance in peripheral tissues such as skeletal muscle and adipose tissue itself, constituting an early defect in the pathogenesis of type 2 diabetes [15]. Different research strategies revealed the complexity of the adipocyte secretome, and to date more than 600 potentially secretory proteins were identified [2].

In addition, it is well accepted that contracting skeletal muscle secretes enhanced levels of myokines which have a beneficial endocrine effect on other organs, presenting novel targets for the treatment of metabolic diseases and type 2 diabetes [16].

## 2. Identification of Contraction-Regulated Myokines

It is well accepted that physical activity exerts multiple beneficial effects on the prevention of chronic diseases, both due to an improved energy balance and due to effects independent of obesity. It is assumed that contraction-regulated myokines play a pivotal role in the communication between muscle and other tissues such as adipose tissue, liver, and pancreatic cells [16–18].

Research of the last decade revealed that several myokines are regulated by contraction, like angiopoietin-like 4 (ANGPTL4), brain-derived neurotrophic factor (BDNF), fibroblast growth factor (FGF) 21, follistatin-like 1 (FSTL1), interleukin (IL)-6, IL-7, IL-15, irisin, leukemia inhibitory factor (LIF), myonectin, myostatin, and vascular endothelial growth factor (VEGF) (for references see Table 1). For some of these reported myokines, the description as a myokine is based on mRNA data of skeletal muscle biopsies. For example, Matthews et al. [19] report increased BNDF mRNA level in human contracting skeletal muscle biopsies. Although the authors could prove enhanced serum levels after exercise in humans and increased BDNF protein level after electrical pulse stimulation of C2C12 cells, BDNF basal secretion could not be detected in the media from skeletal muscle cells *in vitro* [19]. Secretion is the critical characteristic of a myokine and it is preferable to restrict the term myokine to those proteins that are released by skeletal muscle cells themselves.

Nevertheless, the term myokine has also been employed to describe a protein that is synthesized by skeletal muscle tissue, rather than by the skeletal muscle cell. The initial characterization of a candidate myokine is frequently the detection of the gene expression in skeletal muscle tissue by mRNA expression or immunodetection of protein lysates. One dilemma in only determining gene expression or protein level in skeletal muscle biopsies is that aside from skeletal muscle fibres, skeletal muscle contains extended layers of connective tissues, capillaries, and nerve cells among others. Thus satellite cells, endothelial cells, fibroblasts, and motor neurons are included in the analysis. Gene expression must be followed by the detection of the encoded protein in skeletal muscle fibers. Additional immunostaining of the skeletal muscle tissue sections shows that the protein production is indeed intramyocellular. When the expression is first identified in skeletal muscle tissue, the validation of a protein as a myokine has to include that secretion from skeletal muscle cells is demonstrated. In practice, this will generally reflect selective release from skeletal muscle cells *in vitro* either by the use of primary human or animal skeletal muscle cells or from clonal cell lines. Equally, proteins that have been identified in skeletal muscle cells needs to be verified for the native tissue. We recommend that the term myokine is used for a protein that is synthesized and secreted by skeletal muscle cells.

The identification of a protein as a contraction-regulated myokine represents an additional critical step in the analysis. Repeated biopsy sampling from one muscle is necessary to investigate muscular adaptation to different forms of exercise. The adaptation is thought to be the result of cumulative effects of transient changes in gene expression in response to single exercise bouts. Nevertheless, it was shown that multiple fine needle biopsies obtained from the same muscle region can per se influence the expression of marker genes induced by an acute bout of resistance exercise [20]. Thus, repeated biopsies have to be taken carefully in regard to avoiding an inflammatory response in the tissue. In the case that contraction regulation of a protein is first identified in muscle biopsies on the mRNA level, it is essential to determine whether the enhanced mRNA expression is translated to enhanced protein level. An additional elegant approach is to induce contraction of human skeletal muscle cells or clonal cell lines by electrical pulse stimulation [21–24]. The potentially contraction-regulated myokine can be analyzed on the mRNA and protein level. Most importantly, enhanced secretion can be determined in the supernatants by immunodetection.

## 3. Secretome of Muscle Cells

To gain a broader view, recent efforts have focused on exploring the complete secretome of skeletal muscle by proteomic studies. New technological advances, like array studies and proteomic analysis, made the analysis of the qualitative and quantitative analysis of the secretome of skeletal muscle possible. These approaches extended the list of described myokines rapidly. Chan et al. and Henningsen et al. have investigated altered regulation of secretome components at different time points of muscle differentiation of murine C2C12 cells by a quantitative proteomics approach [25–27], while Yoon et al. have studied the regulation of myokine secretion by rat skeletal muscle cells after insulin stimulation [28] and TNF*α* treatment [29]. Recently, Hittel et al. have explored the secretome from cultured myotubes derived from extremely obese compared with healthy nonobese women [30]. All these studies found hundreds of secreted proteins from skeletal muscle, some regulated by insulin or TNF*α*, others during differentiation. A drawback of all these studies is the use of noncontracting cells although contraction is a major characteristic of skeletal muscle activating intracellular signalling pathways, changing the secretory profile, inducing metabolic adaption and the change of its plasticity. To overcome this problem, Norheim et al. combined the proteomic analysis of the secretome of human myotubes with mRNA expression data of muscle biopsies in response to strength training. Using this approach the authors identified 15 novel contraction-regulated myokines [31]. Recently, Catoire et al. described the effect of endurance exercise on gene expression in exercising and nonexercising human muscle by one-legged cycling [32]. Noticeably, acute exercise also caused substantial gene expression changes in nonexercising leg [32]. This effect might be mediated by changes in circulating factors such as free fatty acids, adrenalin, and lactate, but might also support the myokine concept.

Nevertheless, all these studies indicate that skeletal muscle cells are, like adipocytes, major secretory cells. Clustering these skeletal muscle-derived proteins according to their postulated function revealed that these myokines can be sorted to several groups, including myokines contributing to energy metabolism, angiogenesis, blood vessel regulation, and myogenesis [33].

## 4. IL-6: The Best Characterized Myokine

Some of the first reports in this research field identified IL-6 as a secreted protein from skeletal muscle [34, 35]. The identification that contracting human skeletal muscle releases significant amounts of IL-6 into the circulation during prolonged single-limb exercise was a milestone in this research field and identified skeletal muscle as an endocrine organ [36]. Up to now, IL-6 is the most prominent muscle-derived protein, which was demonstrated to be upregulated in plasma after exercise without muscle damage [34, 37]. The level of circulating IL-6 increases in an exponential fashion in response to exercise [36, 38, 39] and declines in the postexercise period [40]. The magnitude by which plasma levels increase is related to exercise duration, intensity, and the muscle mass involved in the mechanical work [36, 38, 39, 41]. However, during one year of training intervention plasma levels of IL-6 remained unchanged [42]. Plasma IL-6 levels can increase up to 100-fold in response to exercise although less strong effects are more frequent [43]. In addition to human serum and skeletal muscle biopsy data, IL-6 has been shown to be secreted by primary human skeletal muscle cells *in vitro* and its secretion was increased by contraction [24].

## 5. IL-15: A Contraction-Regulated Myokine?

IL-15 is discussed as a contraction-regulated myokine in the literature which may play a role in muscle-fat crosstalk [44, 45] mediating some of the beneficial effects of physical activity [46]. Until today, five groups analyzed the regulation of IL-15 after different exercise protocols in humans. However, conflicting data are published whether physical activity affects IL-15 expression, protein level, and secretion from skeletal muscle.

In a first report, no change in IL-15 mRNA level was described in human *vastus lateralis* muscle biopsy samples, which were taken immediately after 2 h intensive resistance training [47]. Although, Nielsen et al. observed that IL-15 mRNA content was upregulated twofold in human *vastus lateralis* muscle 24 hours following a single bout of resistance exercise, this increase in mRNA level was not accompanied by an increase in muscular IL-15 protein level or plasma IL-15 [48]. In addition, Riechman et al. demonstrated that immediately after the end of one resistance exercise bout, plasma IL-15 increased slightly (approximately 5%) [49]. Recently, it has been shown that 30 min treadmill running at 70% of maximum heart rate resulted in a significant increase in circulating IL-15 level in untrained healthy young men (about 12%), measured 10 min after exercise [50]. Different from these acute exercise studies, Yeo et al. described that both 8-week moderate- and high-intensity resistance exercises enhanced IL-15 serum levels [51]. In this study, the authors showed that IL-15 blood level was significantly enhanced after 8 weeks of moderate intensity resistance exercise (250%), while the increase of the myokine prototype IL-6 was rather small (115%). High intensity resistance training also enhanced IL-15 blood levels, but to a lower extent (150%).

In addition, training studies were performed in mice and rats. It has been shown that IL-15 mRNA expression in *soleus* and *gastrocnemius* muscle is increased after 8-week treadmill running training in rats, while plasma IL-15 level was not changed [52]. Yang et al. observed about 1.7-fold increase in IL-15 mRNA expression after three weeks of free wheel running in mice but did not analyze protein or serum level.

Nevertheless, while the observed increase in plasma IL-15 levels in humans is rather small (5–12%) after acute exercise and not depending on the mode of exercise, particularly moderate intensity resistance exercise had a significant effect on IL-15 blood levels. However, to the best of our knowledge, secretion of IL-15 from muscle cells has not been described yet, and it has not been shown that the observations on muscle mRNA level are translated to meaningful contributions to IL-15 serum levels.

## 6. Irisin: A Novel Myokine

Just recently, a novel identified myokine has drawn the attention as a novel preventive and therapeutic target to treat obesity and metabolic diseases like type 2 diabetes. Bostrom et al. observed that overexpression of PGC1*α* in mice muscle as well as exercise induces the expression of the FNDC5 (fibronectin type III domain containing protein 5) gene, a gene which has scarcely been studied before. FNDC5 is described as a protein containing a signal peptide, fibronectin type III repeats, and hydropathy analysis revealed a hydrophobic region, which is likely to encode a transmembrane domain. Previous studies linked the gene to differentiation of myoblasts and neurones [53, 54], and it has been suggested that FNDC5 is located in the matrix of peroxisomes [53]. Bostrom et al. described that the C-terminal tail of the protein is located in the cytoplasm, whereas the extracellular N-terminal part is supposed to be cleaved and released as novel messenger molecule called irisin [55]. Mice subjected to three weeks of free wheel running showed enhanced muscle mRNA expression and elevated irisin plasma concentrations (65%). In addition, ten weeks of supervised endurance exercise training revealed a twofold increase in circulating irisin levels compared to the nonexercised state in a cohort of older subjects [55]. Both the mice and human study analyzed the long-term effect of exercise on irisin plasma levels. It is not described if enhanced serum levels of FNDC5 after muscle contraction are dependent on enhanced gene expression or dependent on enhanced cleavage of the membrane protein.

However, using gene-chip probe sets Timmons et al. observed no effect on FNDC5 mRNA level neither after 6 weeks of intense endurance cycling in younger subjects nor after supervised resistance training [56]. Timmons et al. demonstrated that FNDC5 induction in muscle occurred only in highly active elderly subjects compared to sedentary controls (1.3fold), which were a minority of analyzed subjects. They failed to confirm FNDC5 gene expression by aerobic exercise in younger subjects. Huh et al. observed minor effects on irisin plasma levels after 1 week of exercise (increase of about 18%) and no effect after prolonged training over 8 weeks [57]. Up to now, there is only one study showing a robust activation of FNDC5 after exercise in humans measured by RT-PCR in muscle biopsies [24], however, limited to a very small number of subjects.

Although Bostrom et al. described the discovery of the novel myokine irsin, the authors showed that the release of irisin exclusively in HEK 293 cells transfected with a vector expressing FNDC5 followed immunodetection of culture media protein. It has not been confirmed that muscle FNDC5 mRNA is translated to protein in primary or clonal skeletal muscle cells, and, most importantly, it has not been shown that irisin is secreted from skeletal muscle cells. Furthermore, to demonstrate the secretion of irisin from HEK293 cells and to analyze murine and human serum samples, the authors used an antibody, which most likely cannot detect the cleaved irisin, since the antibody used is directed against the C-terminal part of the protein (Abcam 149–178, C-terminal) [58]. Taken together, future studies should address FNDC5/irisin precise expression and cleavage mechanism to clarify the controversy of current literature.

## 7. BDNF: Released from the Muscle or the Brain?

BDNF belongs to the family of neurotrophins (NT), which includes nerve growth factor, BDNF, NT-3, NT-4/5, and NT-6. These proteins are produced as large precursor proteins that are then cleaved to form the mature neurotrophic protein (reviewed in [59]). In the literature BDNF is discussed as a contraction-regulated myokine [6]. During myogenic differentiation, the expression of BDNF is drastically reduced and is hardly detectable in adult rat skeletal myofibers [60]. By reverse transcription PCR, *in situ* hybridization, and immunofluorescence, it was shown that BDNF is not expressed at significant levels within mature myofibers [60]. *In situ* hybridisation analysis revealed that in adult rat muscle the constitutive expression of muscular BDNF is confined to the myofibres. Satellite cells, Schwann cells, endothelial cells, fibroblasts, or axons did not appear to contribute to BDNF production in normal muscle [61]. In complementary cell culture experiments, it has been shown that levels of BDNF correlate with the population of satellite cells [60]. BDNF is required for early phases of myogenic differentiation, which is delayed in the absence of BDNF [62]. Nevertheless, BDNF protein level was determined in lysates of rat L6 cells [60] and murine C2C12 cells [19].

Serum BDNF levels increased after a graded cycling exercise test in humans (30%) [63] and at the point of exhaustion at the end of a ramp test (about 25%) [64]. Matthews et al. reported enhanced BDNF mRNA and protein expression in human skeletal muscle and after bicycle exercise [19]. Cell culture experiments using murine C2C12 cells stimulated by electrical pulse stimulation confirmed that contractile activity enhanced BNDF mRNA and protein level. Although Matthews et al. report increased serum levels after exercise in humans, BDNF basal secretion by C2C12 that underwent contraction has not been proven and overexpression of BDNF in mouse skeletal muscle did not lead to differences in plasma BDNF [19], leading the authors to the conclusion that BNDF exerts its action locally and is not released into the circulation. Matthews et al. reported an autocrine effect of BNDF since treatment of skeletal muscle cells with recombinant BDNF resulted in enhanced phosphorylation of AMP-activated protein kinase (AMPK) and ACC in rat L6 cells which leads to enhanced fatty acid oxidation [19].

In mice, treadmill exercise induced an increase in BDNF mRNA expression in the hippocampus and cortex (three- to fivefold) [65]. In humans, a BDNF release from the brain was observed at rest and increased two- to threefold during exercise. Both at rest and during exercise, the brain contributed 70–80% of circulating BDNF [65]. These results suggest that the brain is a major, but not the sole contributor to circulating BDNF after exercise.

## 8. MCP-1

MCP-1 is a chemokine and member of the small inducible cytokine family. It plays a crucial role in the recruitment of monocytes and T lymphocytes into tissues [66, 67]. MCP-1 was detected in supernatants of C2C12 cells [68]. In mice, plasma IL-6 levels were markedly increased 3 h following maximum progressive swimming, while MCP-1 plasma levels were not altered by exercise [69]. However, a single bout of intense resistance exercise increased MCP-1 mRNA expression in muscle biopsy samples obtained from *vastus lateralis* muscle about 35-fold after two hours. In comparison, IL-6 mRNA expression, the myokine prototype, was enhanced about 400-fold [70]. One bout of moderate-intensity cycle exercise increased MCP-1 mRNA levels in *vastus lateralis* muscle biopsy samples after 40 min [71]. One-legged cycling of male subjects induced a significant change in MCP-1 mRNA levels in the exercising leg and enhanced MCP-1 plasma levels after exercise and after 3 hours of recovery [72]. In addition, increased MCP-1 mRNA expression in skeletal muscle was reported in elderly individuals following one bout of resistance exercise [73] and in young men after a repeated eccentric exercise bout [74]. Immunohistochemistry analysis of muscle biopsies colocalized MCP-1 with resident macrophage and satellite cell populations, suggesting that alterations in cytokine signalling between these cell populations may play a role in muscle adaptation to exercise [74].

## 9. ANGPTL4: Regulated by Free Fatty Acids

ANGPTL4 represents a prominent long chain fatty acid-responsive gene in human myotubes. Kersten et al. reported that plasma ANGPTL4 levels in humans increased significantly in response to long-term fasting, chronic caloric restriction, and endurance training. All these states are characterized by enhanced circulating FFA [75]. Fasting plasma ANGPTL4 levels of healthy, untrained male volunteers increased during endurance exercise at 50% VO_2_ max for 2 h and especially during subsequent recovery. Importantly, the increase in plasma ANGPTL4 was abolished when subjects were given oral glucose, which induces insulin release and thereby suppresses plasma FFA levels [75]. While ANGPTL4 is below the detection limit in supernatants of differentiated C2C12 cells, long-term treatment of human myotubes (48 h) with the PPAR*δ*-specific activator GW501516 results in the accumulation of ANGPTL4 in the supernatant [76]. In addition, incubation of human primary myocytes with oleic acid and linoleic acid enhanced ANGPTL4 mRNA expression. Nevertheless, this effect was not only observed in primary human myocytes, but also in FAO hepatoma cells and mouse intestinal MSIE cells [75].

Catoire et al. have shown in a human one-legged exercise study that target genes of PPAR transcription factors including ANGPTL4 were induced equally in exercising and nonexercising muscle [77]. Although PPAR*δ* is known to be activated by high-intensity exercise [78], Catoire et al. have concluded that the increase of plasma free fatty acid levels due to acute exercise activates PPARs and therefore ANGPTL4 [77].

Long-term changes in plasma ANGPTL4 levels are most likely mediated by changes in plasma free fatty acids, which raise ANGPTL4 gene expression in target tissues. Nevertheless, ANGPTL4 is ubiquitously expressed in human tissues and highest expression levels were found in liver, followed by adipose tissue, thyroid, brain, small intestine, and less in skeletal muscle [75]. Thus, skeletal muscle might not be the only tissue which is responsible for enhanced ANGPTL4 plasma levels in states of increased FFA levels like endurance training. Furthermore, ANGPTL4 stimulates adipose tissue lipolysis, leading to elevation of plasma free fatty acid levels. Kersten et al. speculated that both mechanisms operate as a positive feedback loop. Free fatty acids raises plasma ANGPTL4 and ANGPTL4 raises plasma free fatty acids by the stimulation of adipose tissue lipolysis [75].

## 10. FGF21

FGF21 is a member of the fibroblast growth factor super family, a large family of proteins involved in cell proliferation, growth, and differentiation. The first evidence that FGF21 is an Akt-regulated myokine was published by Izumiya et al. [79]. FGF21 protein expression and secretion are upregulated by insulin and inhibited by PI3-kinase inhibitor in cultured C2C12 myocytes [79]. Skeletal muscle mRNA level and plasma level are induced by hyperinsulinemia studied in young healthy men during a hyperinsulinemic-euglycemic clamp [80]. In line with this observation, circulating FGF21 is elevated in impaired glucose tolerance and type 2 diabetes patients and correlates with muscle and hepatic insulin resistance [81].

Interestingly, an acute bout of treadmill exercise did not change FGF21 serum levels in sedentary young women. However, after two weeks of exercising there was a 1.6-fold increase in serum FGF21 [82]. In contrast, twelve-week exercise program combining aerobic and resistance exercise, five times per week, reduced FGF21 plasma levels in nondiabetic, obese women (from 230.2 ± 135.9 versus 102.6 ± 117.8 pg/mL) [83]. Nevertheless, nothing is known about the acute effect of contraction on the expression, protein level, and secretion of FGF21 from skeletal muscle cells.

## 11. Myonectin

Myonectin belongs to the C1q/TNF-related protein family (C1QTNF isoform 5) and shows a sequence homology with adiponectin in the shared C1q domain, the signature that defines this protein family [84]. Before myonectin was described as a myokine, the protein was reported to be expressed in the retinal pigment epithelium, and mutations in this gene caused abnormal high molecular weight aggregate formation, which results in late-onset retinal macular degeneration in humans [85].

Myonectin is supposed to be myokine due to the detection of the myonectin transcript in mice skeletal muscle, with significantly lower expression in other tissues, immunoblot detection of myonectin in mouse skeletal muscle lysates [86], and L6 supernatants [87] as well as induced expression during differentiation of mouse C2C12 cells [86].

Currently, one human and one mice exercise studies report divergent results regarding the regulation of myonectin by contraction. Lim et al. reported that a 10-week exercise training program in younger and older groups of healthy women decreased significantly myonectin serum levels, while training increased VO_2_ max, mitochondrial DNA density in skeletal muscle, and plasma adiponectin levels significantly [88].

On the other hand, free wheel running for two weeks increased myonectin expression in soleus und plantaris muscle of mice and circulating serum levels, suggesting a potential role of myonectin in exercise-induced physiology [86]. Recombinant myonectin induced the phosphorylation of AMPK, leading to increased cell surface recruitment of GLUT4, enhanced glucose uptake, and stimulated fatty acid oxidation [87]. Thus, enhanced myonectin secretion induced by contraction could activate signaling pathways providing enhanced energy demands during contraction.

Most intriguingly, Seldin et al. reported that recombinant myonectin promotes fatty acid uptake in mouse adipocytes and rat hepatocytes *in vitro* by enhancing CD36, FATP1, and Fabp4 mRNA expressions, which are known to play important roles in fatty acid uptake. In addition, recombinant myonectin had no effect on adipose tissue lipolysis [86]. A question that should be addressed by future studies is why physical activity activates the secretion of a myokine that induces fatty acid uptake by adipose tissue. Enhanced myonectin serum levels after exercise would therefore lead to an endocrine signal that would deplete energy sources in the blood which is needed by the exercising muscle. On the other hand, long-lasting increase in myonectin serum levels could increase fatty acid uptake in adipose tissue and therefore improve fat metabolism and lipid handling.

## 12. Adipo-Myokines

In a recently published review, Pedersen and Febbraio suggest that skeletal muscle might mediate some of the well-established protective effects of exercise via the secretion of myokines that counteract the harmful effects of proinflammatory adipokines [6].

**Figure 1:**
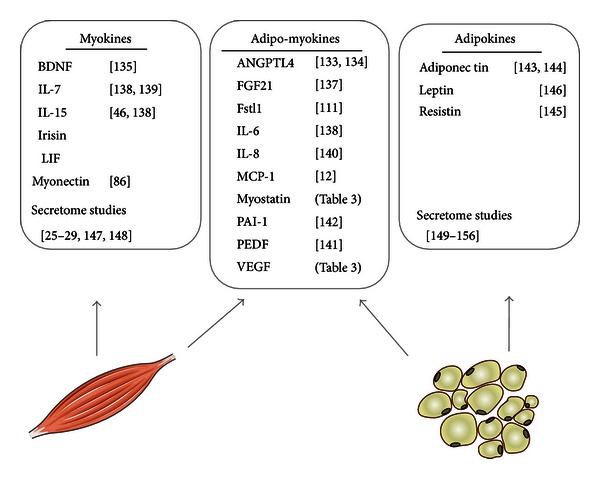
Adipokines, myokines, and adipo-myokines. A search of original articles in pubMed was performed for all myokines described in Table 1 to identify myokines that were also secreted by adipocytes. The search terms used were “adipose tissue,” “adipocyte,” and the indicated cytokine/protein. Reference lists of identified articles were also used to search for further papers. Indicated references published data that the cytokines/proteins are secreted or not secreted by adipocytes or adipose tissue. No data was published on the secretion of LIF and irisin from adipocytes or adipose tissue.

Table 1 summarizes the most prominent myokines, which are described to be contraction regulated. For more than half of the described myokines, ten out of seventeen, secretion by adipocytes has also been described (Figure 1). We termed these cytokines adipo-myokines. How should a cytokine exert on the one hand inflammatory signalling in the obese state and have beneficial effects after exercise? Is it likely that a bidirectional communication between fat and muscle cells takes place? Just recently, Christiansen et al. reported that acute exercise increases circulating inflammatory markers in overweight and obese compared with lean subjects [89]. Why should inflammatory markers increase after exercise?

## 13. IL-6: The Prototype Adipo-Myokine

IL-6 seems to be a good example for an adipo-myokine that is released by both tissues and has the potential to act on both tissues. As described before, the level of circulating IL-6 increases after an acute bout of exercise in an exponential fashion [36, 38, 39] and declines in the postexercise period [40].

In addition, the quantitative release from adipose tissue correlates positively with increased body fat content, which results in systemic elevation of IL-6 plasma levels [90]. It is overexpressed in human fat cells from insulin-resistant subjects [91], increased in the plasma of obese patients [92, 93], and associated with type 2 diabetes [90, 94], while it was described to be decreased after bariatric surgery [95]. IL-6 expression was known to be activated by proinflammatory IKK*β*/NF*κ*B signalling pathway which is thought to contribute to the development of obesity-induced insulin resistance [96, 97]. In addition, it has been shown to inhibit insulin-signalling pathways in the liver [98, 99] and adipocytes [91]. *In vitro* experiments revealed that IL-6 induced insulin resistance in hepatocytes [99], adipocytes [91], and in skeletal muscle cells after treatment with high doses [100]. Incubation of the rat L6 myotubes with 200 ng/mL recombinant IL-6 induced insulin resistance on the level of diminished Akt phosphorylation after 96 h [101] and in primary human myotubes after 48 h [100].

Since exercise is thought to increase insulin sensitivity, the observation that IL-6 is also increased after exercise seemed quite paradoxical. Interestingly, for skeletal muscle cells, *in vitro* studies showed that a rather brief challenge of minutes to few hours with recombinant IL-6 had a positive autocrine effect on skeletal muscle cells. Recombinant IL-6 enhanced insulin-stimulated Akt phosphorylation in primary human myotubes (20 ng/mL) [102, 103] and rat L6 myotubes (about 200 ng/mL) [101]. In addition, basal and insulin-stimulated glucose uptake and translocation of GLUT4 to the plasma membrane were enhanced after 5–120 min in L6 myotubes (1–100 ng/mL) [104]. Furthermore, IL-6 rapidly and markedly increased AMPK and increased fatty acid oxidation [104, 105]. Interestingly, the regulation of intracellular signalling mechanisms, mediating IL-6 expression, differs from the classical proinflammatory pathway. IL-6 expression in contracting muscle is regulated by c-Jun terminal kinase (JNK)/activator protein-1 [106] and increases insulin-stimulated glucose disposal in humans and glucose uptake as well as fatty acid oxidation in rat myotubes *in vitro* [104].

Although one study reported that acute IL-6 administration in resting healthy young men in physiological concentrations did not affect whole-body glucose disposal, net leg-glucose uptake, or endogenous glucose production [107], some evidence is published that IL-6 might have systemic effects. IL-6 knock-out mice develop late onset obesity and impaired glucose tolerance [108]. In healthy humans IL-6 infusion increases glucose disposal without affecting the complete suppression of endogenous glucose production during a hyperinsulinemic-euglycemic clamp [104]. This insulin-sensitizing effect of IL-6, without influencing glucose output from the liver, indicates that the main effect of IL-6 on insulin-stimulated glucose metabolism is likely to occur in peripheral tissues and might just affect skeletal muscle itself or adipose tissue as well. IL-6 was described as a potent modulator of fat metabolism in humans, increasing fat oxidation and fatty acid reesterification without causing hypertriacylglyceridemia [109]. Infusion of rhIL-6 in physiological concentrations into healthy humans increased whole-body fat oxidation [109].

In summary, IL-6 is released by contracting human skeletal muscle and seems to have a beneficial effect on insulin-stimulated glucose disposal and fatty acid oxidation after acute stimulation. These findings support the hypothesis that the myokine IL-6 is important for muscle metabolism during contraction, whereas the chronic elevation of IL-6 released from adipocytes may induce insulin resistance. In addition, Weigert et al. propose a different influence of IL-6 depending on the target tissue. In energy-supplying tissues like the liver and fat the insulin signal is attenuated, whereas in energy-utilizing tissues like the skeletal muscle insulin action is improved [102].

## 14. Follistatin Like 1: An Adipo-Myokine

FSTL1 is the smallest member of the SPARC protein family and a secreted glycoprotein of 45–55 kDa that, despite limited homology, has been grouped in the follistatin family of proteins. A proteomic approach found FSTL1 in the supernatant of primary human adipocytes [110]. FSTL1 is highly expressed and secreted in 3T3-L1 preadipocytes and dramatically downregulated early in their differentiation to adipocytes [111]. Nevertheless, subcutaneous white adipose tissue, lung and heart are the primary sites of FSTL1 transcript expression, compared to brown adipose tissue and muscle of adult murine tissues [111]. Three proteomics studies performed in murine C2C12 and rat L6 cell lines identified FSTL1 as a myokine which is secreted by skeletal muscle cells [25, 27, 28]. These studies found that FSTL1 is secreted by murine and rat skeletal muscle cells, and its secretion is decreased during insulin stimulation and myogenesis. In addition, Görgens et al. recently showed that FSTL1 is also secreted by primary human skeletal muscle cells [112]. Data have shown that FSTL1 is secreted into the media by cultured C2C12 skeletal muscle cells, and it can directly act on endothelial cell-signalling pathways that promote function and survival. FSTL1 overexpression in endothelial cells was found to enhance endothelial cell differentiation and migration and diminish endothelial apoptosis [113]. Thus, FSTL1 might be a myokine that mediates some of the well-established protective effects of exercise that counteract the harmful effects of proinflammatory adipokines on the vasculature. In addition, treatment of neonatal rat ventricual cardiomyocytes with recombinant FSTL1 induced a time- and dose-dependent increase in AMPK and ACC phosphorylation [114]. Given that FSTL1 mRNA expression was increased after strength training [31] and after an acute bout of cycling at 70% VO_2_ max [112], it might be speculated that exercise activates FSTL1 expression and secretion in skeletal muscle, which might act in an autocrine and/or endocrine manner and activate muscular and/or adipogenic AMPK. However, the biological role of adipocyte-derived and muscle-derived FSTL1 has still to be defined.

## 15. Leptin: An Adipokine rather than a Myokine

**Figure 2:**
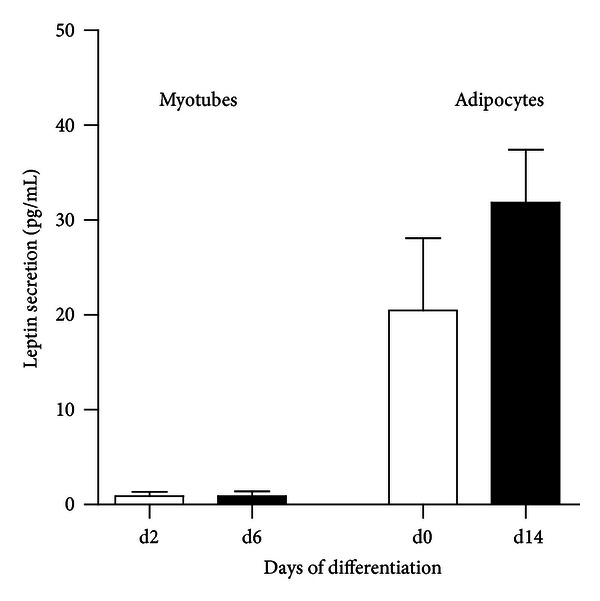
Leptin secretion from primary human myotubes and adipocytes. Primary human skeletal muscle cells and preadipocytes were differentiated *in vitro* for 6 and 14 days, respectively. Supernatants were collected on day 2/6 and day 0/14 of differentiation, respectively. Leptin secretion was measured by ELISA (R&D Systems), *n* ≥ 8. Samples were measured according to the manufacturer's instructions.

Originally, leptin was described as an adipokine which controls food intake [115]. It is synthesized and released in response to increased energy storage in adipose tissue [115–117]. Leptin was detected on the mRNA level in several tissues including skeletal muscle tissue [118]. Recently, Wolsk et al. published that human skeletal muscle releases leptin *in vivo* [119]. The secretion of leptin from skeletal muscle was measured by insertion of catheters into the femoral artery and vein draining the skeletal muscle. The secretion from adipose tissue was measured by an epigastric vein draining the abdominal subcutaneous adipose tissue. The authors measured a leptin release of 0.8 ± 0.3 ng min^−1^ 100 g tissue^−1^ from adipose tissue and 0.5 ± 0.1 ng min^−1^ 100 g tissue^−1^ from skeletal muscle. From these results the authors conclude that the contribution of whole-body leptin production could be substantial greater than skeletal muscle compared to fat due to the greater muscle mass in lean subjects. Earlier work also observed leptin release from human skeletal muscle tissue and subcutaneous adipose explants, with more than ten times less secretion from muscle explants compared to fat explants [120]. Here, we measured the leptin release of differentiated primary human adipocytes and myotubes. The myotubes do not secrete leptin or at levels close to the detection limit (Figure 2). Even in concentrated supernatants of myotubes leptin secretion was barely detectable and contraction induced by electrical pulse stimulation had no effect on leptin secretion (Raschke, unpublished observation). Nevertheless, increasing evidence revealed inter- and intramuscular accumulation of nonmyogenic cell types, which might contribute to the observed leptin secretion of skeletal muscle tissue. Preadipocytes of unknown origin are observed in skeletal muscle [121, 122], and macrophage accumulation is slightly enhanced in skeletal muscle in obese type 2 diabetic subjects or in elderly individuals [123]. From the current data, we would conclude that leptin is rather a true adipokine, instead of an adipo-myokine.

## 16. IL-15: A Myokine rather than an Adipokine

Besides BDNF, IL-7, irisin, LIF, and myonectin, IL-15 is a myokine, which is mainly expressed in skeletal muscle and not in adipose tissue. The most prominent effect of exercise on IL-15 serum levels was observed after moderate intensity resistance training [51]. IL-15 mRNA level in muscle biopsies taken from marathon runners increased more compared to other cytokines like IL-6, IL8, and TNF*α* [47]. Most interestingly, it is higher expressed in skeletal muscle compared to adipocytes [46], while most adipo-myokines are higher expressed in adipocytes compared to myotubes (Table 3). Since IL-15 has been described to have anabolic effects, it may play a role in reducing adipose tissue mass as part of muscle-adipose tissue crosstalk [124]. In 3T3-L1 adipocytes, the administration of IL-15 inhibited lipid accumulation and stimulated secretion of the adipocyte-specific hormone adiponectin [46]. In addition, IL-15 overexpression in mice promotes endurance and oxidative energy metabolism and enhances exercise-related transcription factors in muscle [125] and most interestingly IL-15 treatment improves glucose homeostasis and insulin sensitivity in obese mice [126]. In human subjects, negative correlations between circulating IL-15 levels and both total and abdominal fat have been demonstrated [127]. Since both IL-15 and physical exercise have positive effects on body composition, IL-15 is discussed as a contraction-regulated myokine in the literature which may play a role in muscle-fat cross-talk [44, 45] mediating some of the beneficial effects of physical activity. Yang et al. published just recently a direct link between treadmill exercise of high-fat diet rats, enhanced expression of IL-15 in muscle, and increased IL-15 receptor alpha expression in adipose tissue [52].

## 17. Other Adipo-Myokines

MCP-1 is one of these adipo-myokines. Before MCP-1 was characterized as a myokine, it was described to be produced in isolated adipocytes, associated with adiposity and reduced after weight loss in morbid obese subjects [128]. It is overexpressed in obese rodents [129, 130] and reaches significantly higher plasma levels in diabetic patients [131]. In addition, MCP-1-induced macrophage infiltration in adipose tissue leads to a chronic state of low-grade inflammation [132], which is linked to insulin resistance. *In vitro* data demonstrate that this factor has the ability to induce insulin resistance in adipocytes and skeletal muscle cells [12].

Nevertheless, while IL-6, IL-7, IL-8, MCP-1, and pigment endothelial derived factor (PEDF) are associated with obesity and insulin resistance, these proteins are contraction-regulated myokines (Table 1), and only for IL-6 a beneficial effect has been described (Table 2). The description of a beneficial effect for these myokines is lacking and is an interesting open question for future studies (Table 2). Myostatin is a well-known myokine, but our group recently identified myostatin as an adipokine [2].

## 18. Conclusion

Taken together, one protein can be a myokine as well as an adipokine, indeed two sides of the same coin. In healthy, normal weight subjects skeletal muscle is the largest tissue in the human body, accounting for 40–50% of total human body mass, while body fat accounts for 20–35%. In obese subjects the percentage of total body fat increases to 40–60% resulting in an increased secretion of proinflammatory adipokines, while the percentage of proteins secreted from skeletal muscle during a sedentary lifestyle is decreased. However, for many adipo-myokines the local tissue concentration may be divergent from the serum level, and substantial differences between auto- and endocrine effects of these molecules need to be considered.

As Paracelsus (1493–1541) already coined the famous phrase; “dosis sola facit venenum,” “Only the dose makes the poison”. This might also be true for adipo-myokines. Findings support the hypothesis that the myokines are essential for muscle metabolism during contraction, whereas the chronic elevation of adipokines released from adipocytes may induce adverse effects, even leading to insulin resistance.

## Figures and Tables

**Table 1:** Contraction-regulated myokines. A search of original articles in pubMed was performed for all myokines described to identify contraction regulation of a myokine on the level of enhanced muscle mRNA expression and enhanced serum level. In addition, studies describing basal secretion of the indicated myokine from myotubes (*in vitro * studies) are given. The search terms used were “skeletal muscle,” “myokine,” “exercise,” “secretion,” and the indicated myokine. Reference lists of identified articles were also used to search for further papers.

Myokine	Secreted by cells	Enhanced muscle mRNA level after exercise	Enhanced serum level after exercise
ANGPTL4	*✓* [157]	*✓* [32]	*✓* [75]
BDNF	n.d. [19]	*✓* [19]	*✓* [63, 64]
FGF21	*✓* [79]	—	*✓* [83]^#^
FSTL1	*✓* [112, 113]	*✓* [31]	*✓* [112]
IL-6	*✓* [24]	*✓* [158]	*✓* [35]
IL-7	*✓* [159]	*✓* [159]	—
IL-8	*✓* [140]	*✓* [51, 70, 160, 161]	—
IL-15	n.d. [162–164]	*✓* [48, 52, 55] × [47]	*✓* [49–51] × [52, 165]
Irisin		*✓* [55]	*✓* [55, 57] × [56]
LIF	*✓* [166]	*✓* [166, 167]	—
MCP-1	*✓* [68, 140]	*✓* [70, 72]	*✓* [32, 69]
Myonectin	*✓* [86, 87]	*✓* [86]	*✓* [86] [88]^#^
Myostatin	*✓* [147]	*✓* [168–172]^#^	*✓* [173]^#^
PAI-1	*✓* [31]	*✓* [31]	
PEDF	*✓* [31]	*✓* [31]	
VEGF	*✓* [24]	*✓* [174]	*✓* [51]

*✓*: secretion, enhanced muscle mRNA level, or serum level of myokines have been shown in indicated publications. ×: contraction regulation of myokine has not been shown. ^#^Myokine serum levels are described to be decreased after exercise, n.d.: not detected in supernatants of myotubes.

**Table 2:** Overview of selected adipo-myokines which are associated with obesity and insulin resistance.

Adipo-Myokine	Associated with obesity	Associated with insulin resistance/T2D	Associated with improved glucose metabolism
IL-6	*✓* Plasma IL-6 is positively related to fat mass [175], elevated in type 2 diabetics [90, 94]	*✓* IL-6 promotes insulin resistance [91, 98, 99]	*✓* Insulin-sensitizing effect on skeletal muscle [102, 104] increases whole body fat oxidation [109]

IL-7	**?** Increased mRNA level in omental adipose tissue [139] although mice overexpressing IL-7 have reduced adipose tissue mass [177]	n.d.	n.d.

IL-8	*✓* Higher expression in visceral adipose tissue in type 2 diabetics and insulin resistant subjects [91, 178]	*✓* IL-8 plasma levels correlate with measures of insulin resistance [97, 179]	n.d.

MCP-1	*✓* Serum MCP-1 is increased in obesity [129]	*✓* Promotes insulin resistance [12, 129]	n.d.

PEDF	*✓* PEDF serum levels increased in obesity [180, 181]	*✓* PEDF serum levels associated with insulin resistance [180–183], PEDF promotes insulin resistance [141]	n.d.

*✓*: association has been shown in indicated publications; *✓*: contradictory data published; n.d.: not described.

**Table 3:** Concentrations of various factors in conditioned medium from primary human adipocytes and primary human myotubes. Primary human skeletal muscle cells were differentiated for 5 days and primary human preadipocytes were differentiated for 14 days *in vitro* to mature cells. During the last 24 h cells were incubated with serum-free medium to obtain conditioned medium. Concentrations of secreted factors from cells within this conditioned medium were analyzed by enzyme-linked immunosorbent assay. Data are means ± SEM, *n* ≥ 3.

Secreted factor	Concentration in adipocyte-conditioned medium (ng/mL)	Concentration in skeletal muscle-conditioned medium (ng/mL)
Chemerin	2.12 ± 0.3 [141]	0.006 ± 0.001
DPP4	2.19 ± 1.4 [11]	0.69 ± 0.18
IL-6	0.03 ± 0.002 [141]	0.02 ± 0.003
IL-8	0.15 ± 0.04	0.07 ± 0.01
MCP-1	0.35 ± 0.06	0.33 ± 0.08
Myostatin	12.64 ± 4.44	3.44 ± 1.64
PEDF	45.7 ± 0.82 [141]	5.4 ± 0.86
VEGF	0.33 ± 0.09 [141]	0.05 ± 0.03

## Abbreviations

AMPK: AMP-activated protein kinase
ANGPTL4: Angiopoietin like 4
BDNF: Brain-derived neurotrophic factor
DPP4: Dipeptidyl peptidase 4
FGF: Fibroblast growth factor
FNDC5: Fibronectin type III domain containing protein 5
FSTL1: Follistatin-like 1
IL: Interleukin
LIF: Leukemia inhibitory factor
MCP-1: Monocyte chemoattractant protein 1
NT: Neurotrophins
PEDF: Pigment endothelial derived factor
TNF*α*: Tumor necrosis factor *α*
VEGF: Vascular endothelial growth factor.
